# Seasonality of riverine macroplastic transport

**DOI:** 10.1038/s41598-019-50096-1

**Published:** 2019-09-19

**Authors:** Tim van Emmerik, Emilie Strady, Thuy-Chung Kieu-Le, Luan Nguyen, Nicolas Gratiot

**Affiliations:** 1 The Ocean Cleanup, Batavierenstraat 15, 3014JH Rotterdam, The Netherlands; 2 Hydrology and Quantitative Water Management Group, Wageningen University, Wageningen, The Netherlands; 3 CARE, Ho Chi Minh City University of Technology, VNU-HCM, Ho Chi Minh City, Vietnam; 4 University of Grenoble Alpes, Grenoble, France; 5 Aix-Marseille Univ., Mediterranean Institute of Oceanography (M I O), Université de Toulon, CNRS/IRD, Marseille, France; 6 Faculty of Geology and Petroleum Engineering, Ho Chi Minh City University of Technology, VNU-HCM, Ho Chi Minh City, Vietnam

**Keywords:** Environmental sciences, Environmental impact, Hydrology

## Abstract

Marine plastic pollution is an increasing environmental threat. Although it is assumed that most marine plastics are transported from land to the ocean through rivers, only limited data on riverine plastic transport exists. Recently, new methods have been introduced to characterize riverine plastics consistently through time and space. For example, combining visual counting observations and plastic debris sampling can provide order of magnitude estimations of plastic transport through a river. In this paper, we present findings from multi-season measurement campaign in the Saigon River, Vietnam. For the first time, we demonstrate that macroplastic transport exhibits strong temporal variation. The monthly averaged plastic transport changes up to a factor five within the measurement period. As it is unclear what drives the variation in plastic transport, relations between rainfall, river discharge, presence of organic material and plastic transport have been explored. Furthermore, we present new findings on the cross-sectional and vertical distribution of riverine plastic transport. With this paper we present new insights in the origin and fate of riverine plastic transport, emphasizing the severity of the emerging thread of plastic pollution on riverine ecosystems.

## Introduction

Land-based plastics are considered one of the main sources of marine plastics pollution^1^. Globally, rivers are predicted to convey between 0.4 and 2.75 million tonnes of plastic from land to the oceans annually^2,3^. However, modeled riverine plastic emission estimations remain uncertain as field data on plastic emission into the ocean is scarce.

Targeted measurements of riverine macroplastic (>5 cm) transport only started recently. Previous studies used various types of measurement methods, such as nets^4–7^, manta trawls^8^, surface booms^9–11^, or visual counting of floating plastic debris^12,13^ to estimate macroplastic transport in rivers. However, data has been collected inconsistently and reported in different formats, making it difficult to compare plastic transport over time or across rivers. Especially for the planning of prevention and mitigation strategies, additional information on the spatiotemporal variation of riverine plastic transport is crucial. Recent work presented a new methodology for consistent measurements of riverine macroplastics^14^. A combination of visual counting observations and simple sampling of debris is used to characterize riverine plastic transport. The method allows to provide a first order magnitude of the quantity and composition of riverine plastics over time and space.

Most studies on riverine macroplastics focused on rivers with a relatively low predicted plastic emission, whereas the world’s most polluted rivers remain ungauged^15^. Although South-East Asian rivers are expected to contribute considerably to the world’s total plastic emission^2,3^, only few studies have focused on characterizing plastic transport in such rivers. Recent work by Lahens *et al*.^16^ and van Emmerik *et al*.^14^ presented initial assessments of plastic pollution in the Saigon River, Vietnam. The Saigon River traverses the Vietnam’s economic capital Ho Chi Minh City (HCMC; 8.4 million inhabitants^17^) which suffers, like most Southeast Asian mega cities, from a serious solid waste management problem, with 8,175 tons of solid waste generated per day^18^. Furthermore, the Saigon River is estimated to be the 5th most plastic emitting river in Vietnam and the 45th in the world^2^. Additional data in such rivers will shed additional light on the governing drivers of riverine macroplastic transport, its variation over time and space, the accuracy of global modeling efforts and implementation of potential mediation strategies.

In this paper we present a first detailed analysis of a long-term riverine macroplastic monitoring effort. From March to December 2018, measurements of the plastic quantity, composition and characteristics were done. We focus on discussing the monthly variation in the magnitude and polymer composition. In addition, we present an assessment of the cross-sectional and vertical distribution of riverine plastic transport. We combine all observations to arrive at a best estimate of the total macroplastic outflow from the Saigon River in 2018. Our main goal is to provide new insights in the origin and fate of floating riverine macroplastic transport and highlight the severity of riverine plastic pollution.

## Results and Discussion

### Plastic composition

**Figure 1:**
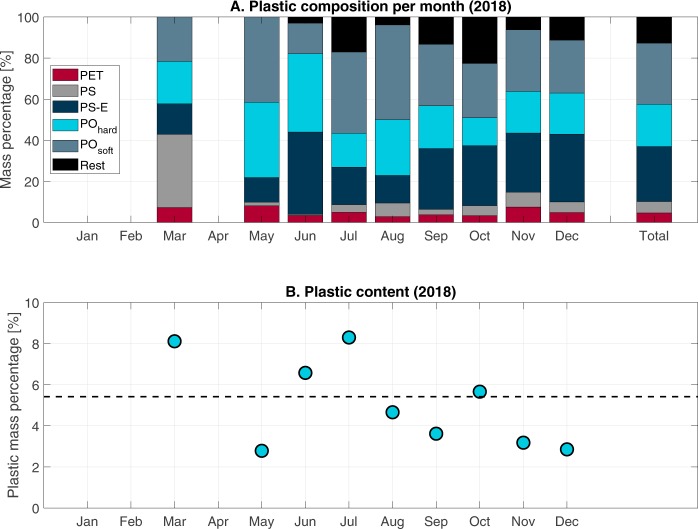
(**A**) Composition of sampled plastic per month, and (**B**) plastic content of total sampled debris per month in 2018, based on the net samples.

**Figure 2:**
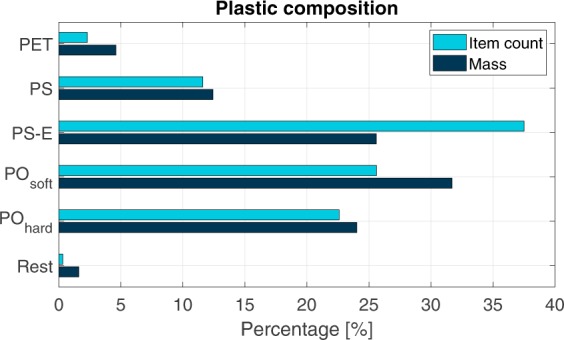
Plastic composition based on mass and item count (N = 3057), determined from net sampling.

Figure 1A presents the monthly composition of wet sampled mass per plastic type. When considering all samples in 2018, PO_soft_ was most abundant (30.6%), followed by PS-E (25.5%) and PO_hard_ (20.6%). PET was the least found plastic type (4.6%) in the Saigon River (Fig. 2). This global plastic composition is in accordance with previous studies on the Saigon River and its urban canals^14,16^, showing the consistency of the floating plastic litter composition throughout the years. The precise composition of the sampled plastic varied from month to month, with most plastic mass being defined as either PO_soft_ (14.8–41.6%) or PS-E (12.0–40.0%). Only in March most plastic was identified as PS (35.7%), see Fig. 1A. We suggest in that case that it might be an artifact of uncertain identification of plastic. It was found that some observers tended to experience difficulties separating PS-E from PS. Plastic composition was determined both in terms as mass percentage as in relative item count. Although in terms of mass PO_soft_ had the largest share (Figs 1A and 2), in terms of item count PS-E was most abundant (38%). PS-E particles were mainly identified as food box fragments^14^. On average, plastic makes up 6% of the floating wet mass (Fig. 1B), which is similar to the estimated fraction by van Emmerik *et al*.^14^ and within the lower estimation range (11–43%) of Lahens *et al*.^16^ in the urban canals of this system. It is also in the same ratio as observed in the Seine River^9^. The wet weight plastic fraction in floating debris is lower than the fraction in the municipal solid waste of Ho Chi Minh City, estimated at 16% [Verma *et al*., 2016]. The share of plastic in total sampled debris mass varied per month, ranging from 3.2 (May) to 8.3% (July). Compared to the Seine River^9^, the main difference of plastic’s type composition is the occurrence of PS/PS-E relative to other plastic types, being proportionally more abundant in the Saigon River. This may be explained by the local habit of street-foods and the extensive use of food packaging for take-away. Note that the plastic fraction was calculated using only the total organic and plastic mass. Other material debris was only measured from March to June, 2018, and was on average 0.9% of the total mass during this period (n = 22 days).

**Figure 3:**
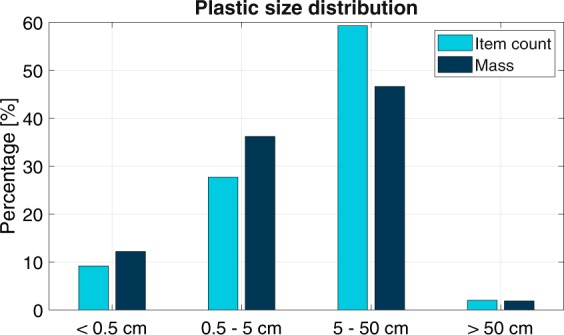
Particle size distribution in respect to cumulative occurrence and cumulative mass (N = 3057), determined from net sampling.

Based on analysis of samples taken on 45 days, the average mass of the sampled plastic items was 1.0 × 10^−2^ kg per item (σ = 3.3 × 10^−2^ kg/item, N = 3057). This is higher than the average mass in previous assessments of the Saigon River (van Emmerik *et al*., 2018), but here only two weeks of sampling was done in March 2018. During this limited sampling period, older and more fragmented plastic may have been present in the river. Figure 3 presents the particle size distribution of the 3057 sampled plastic items, expressed in cumulative occurrence and cumulative mass. A clear majority of the items were in the 5–50 cm range (46% mass count, 59% item count). Recent input from the main source (Ho Chi Minh City) can explain the large item size, as items did not have time to get fragmented yet. This might be biased by the chosen measurement technique, especially the chosen mesh size (see Methods, Table 2). However, in terms of mass it is not expected that additional smaller items will cause a significant shift in the size-mass distribution. Also, observational evidence supports the claim that most plastic items are within the 0.5–50 cm range (82% mass, 87% items). Items smaller than 0.5 cm or larger than 50 cm comprise 9% (items) to 12% (mass) and 2% (items and mass) of the total sampled population, respectively.

The characteristics of the plastic found in the Saigon River shows various similarities and differences compared to marine plastic found in the Great Pacific Garbage Patch (GPGP). For example, in the GPGP barely any foams (PS-E) were found^19^, while in the Saigon River this is the most abundant plastic polymer type in terms of item count, and second in terms of mass. This is explained by the rapid fragmentation of PS-E in aquatic environment, resulting in fewer larger PS-E items making it to the open ocean. For the size classes up to 0.5 m, the distribution of plastic mass is relatively similar to that found in the GPGP. However, a clear difference is the near absence of items larger than 0.5 m (in mass and item count) in the Saigon. In the Pacific Ocean, items larger than 0.5 m accounted for about half of the total plastic mass^19^. Such plastics mostly consist of lines, ropes and fishing nets, which are barely found in the Saigon, and demonstrate that rivers are not the sole sources of plastic litter in the open ocean. The discrepancy between riverine and marine plastics suggests that not all plastic pollution emitted by rivers do not end up as floating items on the open ocean. A considerable amount may in fact wash up ashore, settle on the seabed, or fragment in smaller plastic particles.

### Horizontal distribution

**Figure 4:**
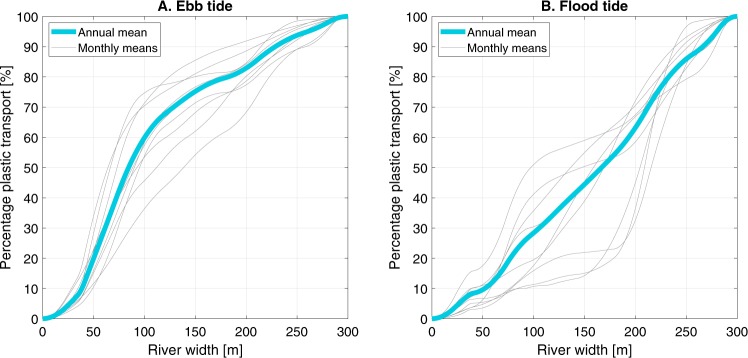
Cross-sectional distribution of plastic transport during (**A**) ebb tide and (**B**) flood tide, based on visual counting.

To assess the variation in horizontal distribution of plastic transport, the observed transport profiles are expressed as percentage of transport per meter river width for both ebb tide (Fig. 4A) and flood tide (Fig. 4B) for each month (see also Fig. S2). On average, the transport during ebb tide has a clear preferential path, as around 60% the transport occurs in 100 m of the river width (300 m in total). Per month, this is subject to change however, varying between 37 and 73% for the same 100 m stretch. The average flood tide flow is more homogenous with, on average, half of the transport passing through half of the river width. A strong variation from month to month was also found here with 10 to 50% of the transport occurring in the same river stretch. The distribution did not show high Pearson’s correlations with tidal range (ebb: r = 0.37, p = 0.33; flood: r = 0.35, p = 0.35), rainfall (ebb: r = 0.11, p = 0.77; flood: r = −0.20, p = 0.61), discharge (ebb: r = −0.42, p = 0.26; flood: r = 0.24, p = 0.54), or the ebb/flood tide time percentage (ebb: r = −0.08, p = 0.0.83; flood: r = 0.53, p = 0.14). We hypothesize that the horizontal plastic distribution is mainly governed by stream flow velocity, wind speed and direction, and navigation. The consistent concentration profile during ebb tide corresponds to the flow velocity profile. During flood tide, this profile seemed to be disturbed by intense navigation. This variable horizontal plastic transport evidences that a single or low number of locations for plastic transport over a river width of 300 m is not enough and it may lead to drastic under- or overestimation. From a remediation perspective, it also points out a challenge for direct plastic retrieval from a river.

### Vertical distribution

**Figure 5:**
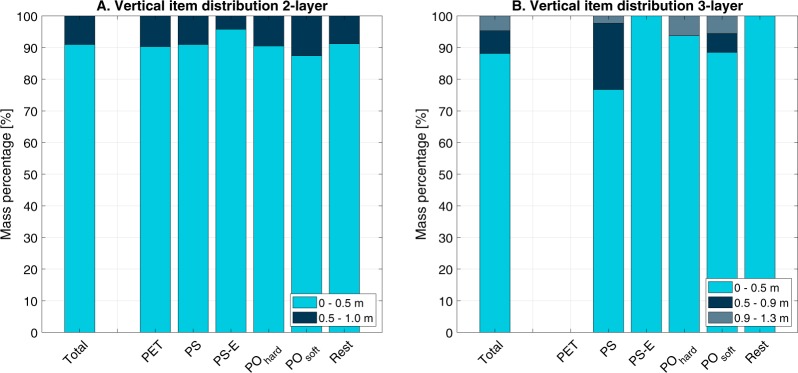
Vertical distribution of plastic items sampled using A. 2- and B. 3-layer trawls.

To transfer the visually counted plastic items per hour into plastic mass, we established an empirical relation between simultaneous visual counting and trawl sampling. From 578 simultaneous visual counting and sampling measurements, we found that each floating particle equals on average 1.9 × 10^−2^ kg (σ = 3.7 × 10^−2^ kg) sampled plastic in the upper 0.5 m of the water column. Note that this is higher than the average mass per individual plastic item (1.0 × 10^−2^ kg/item), as it accounts for all plastics between the surface and 0.5 m depth and not only those detected by visual counting. To estimate the plastic mass transport at deeper layers, additional sampling within the water column was done (Fig. 5). From the measurements using a two-layer net it was found that from all plastics sampled within the upper 1 m of the water column, 91.0% of the items were found in the upper 0.5 m. Using a three-layer net it was found that from all plastics sampled within the upper 1.3 m of the water column, 88.1% was in the upper 0.5 m, 7.2% in the layer from 0.5–0.9 m and 4.7% in the lower layer from 0.9–1.3 m. Some variation between plastic types was found, especially for the three-layer trawl which exhibited mostly PO_soft_ and PO_hard_ in the 0.9–1.3 m layer. For the two-layer net, the distribution was rather equal for most plastic types, with 90% in the upper 0.5 m and 10% in the lower 0.5–1.0 m layer. Only for PS-E (95.8% vs 4.2%) and PO_soft_ (87.4% vs 12.6%) the distribution deviated. PS-E has a density of 640 kg/m^3^ at most, whereas PO_soft_ are closer to being neutrally buoyant and might be more homogeneously distributed over the water column. Furthermore, PO_soft_, often films and foils, have a large surface compared to their volume. The increased sensitivity to turbulence is therefore expected to influence their vertical distribution. In the case of the Saigon River, turbulence can be significant as a result of the tides, bridges and intense shipping traffic. The presence of plastic in both 0.5–0.9 m layer and 0.9–1.3 m evidences that plastic items are sinking independently of their densities as plastic having a lower density than riverine water were sampled below 0.5 m depth, except PS-E at 0.9–1.3 m depth. The development of biofilm on the plastic surface may enhance their density and their ability to settle.

### Monthly variation

**Figure 6:**
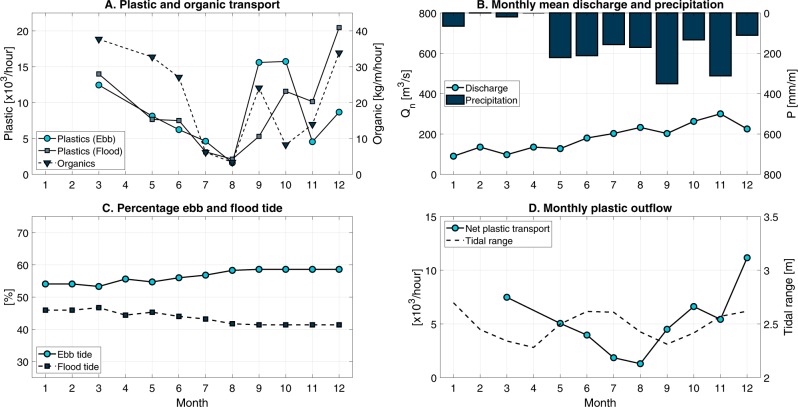
(**A**) Plastic transport and organic debris, (**B**) monthly mean discharge and precipitation, (**C**) percentage ebb and flood tide flow, and (**D**) monthly mean plastic outflow and tidal range.

The measured net plastic flow changed considerably between 4 × 10^3^–19 × 10^3^ items/hour over the sampling period, see Fig. 6D. The abundance of plastics seems to be related (Pearson’s r = 0.65, p = 0.06) to the concentration of organic material in the Saigon River (Fig. 6A). Most organic material was identified as water hyacinths. Water hyacinths often form large patches with plastic floating on its surface or trapped in the roots and thus seem to function as accumulation zones for plastic material. From March to August, the plastic transport decreases steadily, after which it increases again to the annual maximum in December. For this hydrological year, the net water discharge (Fig. 6B) increased gradually from January to December, and therefore does not seem to explain the observed variation in plastic transport (Pearson’s r = 0.04, p = 0.92). Rainfall increased from 0–66 mm/month in January–March to 157–221 mm/month in May–August. The highest rainfall was recorded in September (351 mm/month) and November (312 mm/month), see Fig. 6B. The data suggest no clear relation between rainfall and plastic transport in the Saigon River (Pearson’s r = −0.34, p = 0.34). Using the percentage of incoming (flood) and outgoing (ebb) tide per month (Fig. 6C), the net plastic transport was calculated (Fig. 6D) and varied from 0.4 × 10^4^ items/hour (July, August) to 1.9 × 10^4^ items/hour (December). The variation of monthly mean net plastic transport also does not seem to be related to the annual tidal range variation of the river (Pearson’s r = 0.06, p = 0.89). Within the sampling period, 21% of the monthly transport occurred in December, with July and August contributing the least (4.4% and 4.7%, respectively). As none of the additional data shows a clear relation with the measured plastic transport, the latter is most likely driven by a combination of processes.

### Annual plastic outflow

Based on the monthly average net plastic flow, we present a course estimation of the yearly plastic outflow in terms of mass. Table 1 presents the values of each step in this estimation. We estimate a yearly outflow of 1.1 × 10^3^ t/y floating macroplastics from the Saigon River (1.4–1.6 × 10^3^ t/y if extrapolated to the whole water column). From previous global modeling approaches^2,3^, no estimate for the Saigon River is available. Lebreton *et al*.^3^ estimated that the total emission for the Nha Be river (confluence of Saigon and Dong Nai rivers) is 5.0 × 10^3^ t/y. If distributed proportionally over the catchment areas (Saigon River 23,400 km^2^, Dong Nai River 38,600 km^2^), this Saigon would emit 1.9 × 10^3^ t/y, which is similar in magnitude as our observation based estimate. In our approach, however, we do not take into account microplastics, which is one of the reasons that may explain the difference.

**Table 1:** Yearly emission in items/year and in t/y based on 2018 observations.

Mean hourly emission [n = 578]	Annual transport [floating]	Mass per particle	Annual transport [0–0.5 m]	Vertical distribution upper 1.3 m	Annual transport [0–1.3 m]	Fraction upper 1.3 m	Annual transport [total]
5.7 × 10^3^ items/h	5.0 × 10^7^ items/y	1.9 × 10^−2^ kg/item	9.5 × 10^2^ t/y	88.1% [0–0.5 m] 7.2% [0.5–0.9 m] 4.7% [0.9–1.3 m]	1.1 × 10^3^ t/y	66–79%	1.4–1.6 × 10^3^ t/y

Note that the quantification of several components of the total net transport can be improved. First, there is still a clear challenge for assessing the net water discharge for rivers in tidal environments^20^. Our study uses the first high frequency water discharge estimation for the Saigon River. We recommend to measure plastic transport at multiple locations in future assessments, as that may allow for a mass balance approach. Further work on plastic transport within estuaries will also improve the understanding of how much plastic is emitted into the ocean, and how much is accumulating within the river system.

Second, as the vertical distribution is likely to be influenced by plastic polymer type and flow regimes, this might be different for the Saigon River. As suggested by Morritt *et al*.^6^, a significant share of the total plastic transport might occur at the deeper layers. Data from the Danube River^20^ confirmed this, as 21–34% of the plastic was estimated to be transported below 1.5 m. A general understanding of the vertical distribution of riverine plastics is yet to be established.

Third, the semi-diurnal tidal cycle was found to play a crucial role in net plastic transport and cross-sectional distribution (Fig. 4). As macroplastics may exhibit different mixing behavior than for example sediments or dissolved substances, additional measurements are required to allow for better predictions of net plastic transport. Future assessments might include boat-based manta trawls^21^ or unmanned aerial vehicles^22,23^.

## Conclusions

Plastic transport in the Saigon River exhibits clear seasonality, with a factor of five between measured peak and low monthly emission rates. The peak and low macroplastic transport flow months contribute 21% (December) and 4% (July–August) to the total transport within the measured period, respectively. We estimate the total macroplastic debris outflow in the upper 1.3 m of the Saigon River to be 1.1 × 10^3^ t/y in 2018 (1.4–1.6 × 10^3^ t/y if extrapolated over the whole water column).

For the Saigon River, no clear relation was found between water discharge, rainfall, annual tidal range and plastic transport. We hypothesize that plastic transport is influenced by all those factors and wind, interacting together in a way that require further investigation. We also hypothesize that plastic transport may be strongly linked to the presence of water hyacinths in the river, which act as accumulation zones for plastic items.

Plastic composition changes over time, although PS-E (item count) and PO_soft_ (mass count) are on average the main polymers. The ratio of plastic to organic debris varies with a factor of four over time, which is important for potential prevention and mitigation strategies.

Most plastics (88.1%) were found in the upper 0.5 m of the water column. However, additional measurements are required to further study the vertical distribution along the entire water column. During ebb tide, plastic is distributed ununiformly over the cross section, with 80% of the plastic being transport in 37–73% of the width. However, during flood tide the distribution is more uniformly (80% of the plastic in 70–83% of the width).

With this paper we aim to shed light on the order of magnitude and spatiotemporal variation of riverine plastic transport. Such data are crucial for planning and evaluating future prevention and mitigation strategies. However, our study also emphasizes the need for additional research on the origin and fate of plastics in aquatic ecosystems and its spatiotemporal variation.

## Methods

### Study site

All measurements were taken in the Saigon River in Ho Chi Minh City (HCMC) from 1 March to 13 December 2018, between 07:00 and 17:00. The Saigon River is about 250 km long and has a catchment area of 4717 squared kilometers^16^. The dense urban canal network (700 km) of HCMC drains into the Saigon River. The climate is characterized by a monsoon season from May to October, and a dry season from December to April. Sampling was done from the 300-meter-wide Thu Thiem bridge (10°47′08.3″N, 106°43′06.2″E), located 70 km upstream from the river mouth and 14 km upstream from the confluence with the Dong Nai River. The sampling location was affected by mixed semidiurnal and asymmetric tidal influences, with a tidal range of up to 3 m. Plastic debris counting and sampling was done across the Thu Thiem bridge. More details are found in the Supplementary Materials S1.

### Plastic sampling

Plastic sampling was done using three static bridge-mounted trawls as presented in van Emmerik *et al*.^14,24^. Three different types of trawls were used, varying from one to three layers. The characteristics of the trawls are presented in Table 2. All trawls consist of frames, with 2-meter nets attached. The chosen mesh size was an optimization between the desired size fraction of the plastic catch and the controllability of the trawl due to the drag force. Depending on the water flow velocity, trawling deployments lasted between 1 and 60 minutes. During each trawling experiment, additional visual plastic debris counting was done for the stretch were the trawl was deployed to link the visually observed plastics to the total plastic mass in the upper layer. The trawling location was determined based on the prevailing flow direction and observed largest debris amount. During ebb tide flow trawling was done at location 2 and 3 (Fig. S1), and during flood tide flow trawling was done at location 11 and 12 (Fig. S1). Details on the sampling days per month and the total sampling duration per month can be found in the Supplementary Materials (S3).

**Table 2:** Characteristics of the used trawls.

Trawl	Dimensions [W × H]	Sampled layers	Net mesh size	Sampling period
Surface only	Layer 1: 1 × 0.5 m	1: 0–0.5 m	4 cm	March–December 2018
2-layer	Layer 1: 0.67 × 0.6 m	1: 0–0.5 m	3 cm	July–December 2018
	Layer 2: 0.67 × 0.5 m	2: 0.5–1.0 m		
3-layer	Layer 1: 0.67 × 0.6 m	1: 0–0.5 m	3 cm	July 2018
	Layer 2: 0.67 × 0.4 m	2: 0.5–0.9 m		
	Layer 3: 0.67 × 0.4 m	3: 0.9–1.3 m		

The retrieved samples were divided into three categories: (1) organic, (2) plastic, and (3) other materials. All categorized samples were weighed individually from March to June, 2018. From July to December, only organic and plastic mass was measured. At the end of each day, all plastic samples were visually subdivided into polyethylene terephthalate (PET), polystyrene (PS), expanded polystyrene (PS-E), hard polyolefins (PO_hard_), soft polyolefins (PO_soft_) and rest, and weighed. The samples were sun-dried for at least 24 hours, and subsequently the bulk dry mass was determined for each plastic type. Finally, a subset of samples from 45 days consisting of 3057 plastic items were individually analyzed to determine their mass, size, and plastic type.

### Visual counting

Visual counting of plastic debris was based on the approach presented by González-Fernández & Hanke^12^ and van Emmerik *et al*.^14,24^. At 12 locations across the bridge, the number of plastic pieces that passed through 15-m-wide sections (covering 60% of the cross-section) was visually counted for 2 minutes. The bridge was on average 14 m above the water level. Counting was done hourly facing downstream, as we could identify plastic pieces more accurately. Each floating and superficially submerged plastic piece that was visible was counted, independent of its size. The average minimum debris size was estimated to be 1 cm. If the debris type was uncertain, it was not counted as plastic. For subsequent analysis, the plastic debris counts were normalized over time and distance and expressed in plastic pieces per meter river width, per unit of time. Details on the sampling days per month, total measured profiles per month, and the number of ebb tide and flood tide profiles can be found in the Supplementary Materials (S4).

### River discharge

River discharge estimation was done by applying an adapted Manning-Strickler hydraulic law, as proposed by Camenen *et al*.^25^. This model takes into account the tidal propagation and revealed to be adapted to estuarine environment where tides propagate gently, which is the case in the Saigon River. Two water-pressure sensors were deployed during year 2018 to monitor water level fluctuations every 10 minutes. Two Acoustic Doppler Current Profiler (ADCP) campaigns were performed previously, in September 2016 and March 2017, with a Rio Grande 600 kHz, to calibrate and validate the hydraulic model.

For this study, we use the high frequency data series to calculate monthly mean discharge values. In addition, the monthly mean percentage of outgoing (ebb tide) and incoming (flood tide) flow was calculated. This was done by dividing the total number of positive (ebb tide) and negative (flood tide) values by the total number of observations for each month. The discharge data is presented in the Supplementary Materials (Fig. S5).

### Other data

Monthly precipitation data was used from the Mac Dinh Chi station, located in district 1 of Ho Chi Minh City. Estimated astronomical tide for 2013 and 2018 at Vung Tau, 90 km downstream of The Thiem bridge, was obtained from XTide software. Daily minimum and maximum values were used to compute the daily tidal range, which were averaged per month.

### Estimating net plastic transport

We present two expressions of net plastic transport towards the ocean. First, we estimate the monthly mean transport of floating plastic items into the ocean. Second, we use several assumptions to give an order of magnitude estimation of the total yearly plastic mass flowing into the ocean.

The monthly mean present of plastic items is calculated using the (1) monthly mean outgoing and incoming plastic transport [items/hour] and (2) the monthly mean average percentage of outgoing and incoming flow, expressed as:

$${P}_{n,j}={P}_{j}\cdot {r}_{o,j}$$

With net plastic outflow *P*_*n*_ [items/hour], mean monthly plastic transport *P*_*j*_ [items/hour] and outflowing discharge percentage *r*_*o*_ [−] for month *j*. The outflowing discharge percentage is calculated by counting the total duration of outflowing discharge per month. A two-paired t-test was done using the inflowing and outflowing plastic transport observations, which showed that both populations come from the same distribution with equal mean (5% significance level, p = 0.70), see Fig. S6 in the Supplementary Materials. The mean monthly plastic transport was therefore determined using the absolute values of all plastic transport measurements, regardless of the direction. In our approach we assume that during outflowing plastic transport, plastics are emitted, and do not enter the system after the flow direction reverses.

**Figure 7:**
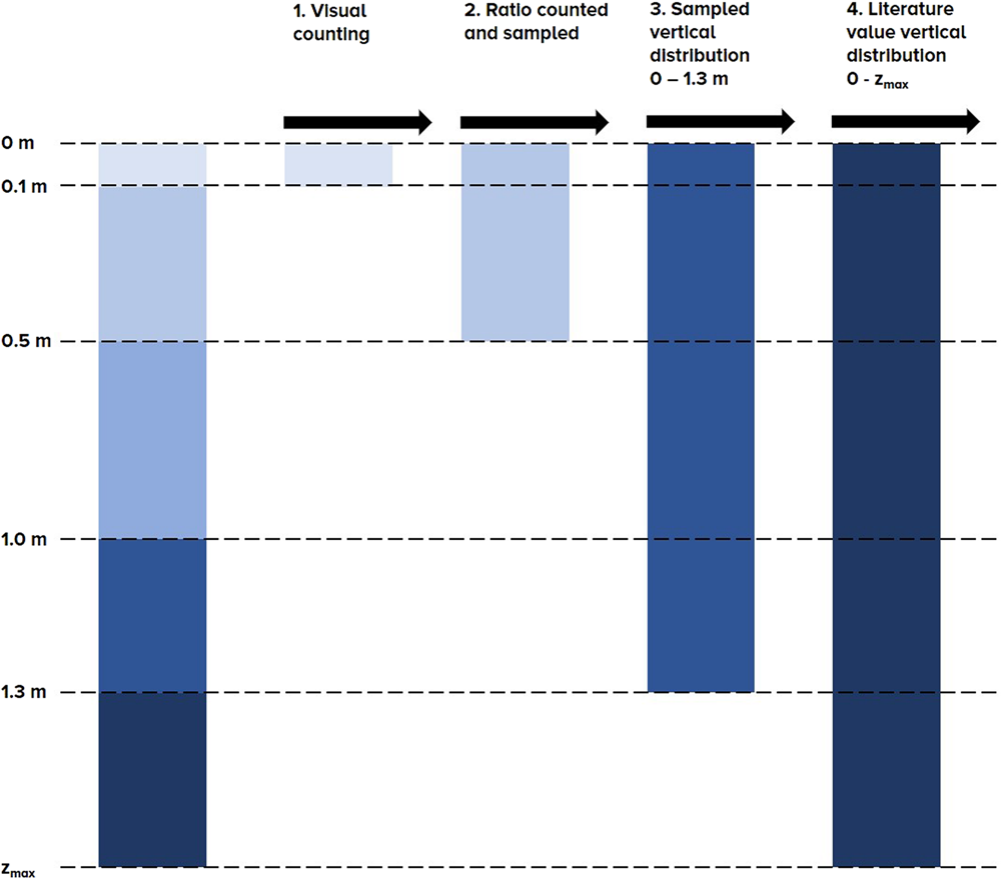
Conceptual model for estimating total plastic transport.

For the estimation of total annual emitted plastic mass, we use the following assumptions (see Fig. 7). First, we calculate the mean hourly plastic item transport from the visual counting and extrapolate it to number of plastic items per year. Second, we use an empirical relation between sampled plastic mass and simultaneously counted floating plastic items to transfer floating plastic items into total plastic mass in the upper 0.5 m (1.9 × 10^−2^ kg/item) Third, we use the total measured vertical distribution of plastic items from the vertical distribution sampling (0–1.3 m) to account for the deeper layers between 0.5 and 1.0 m and between 1.0 and 1.3 m. Finally, to approximate the plastic at lower layers we use the vertical distribution of plastic transport found by Hohenblum *et al*.^20^. Here, it was found that 66–79% of the plastic transport occurs in the upper 1.5 m. We use these values to estimate total plastic transport from the measurements done in the upper 1.3 m in the Saigon River.

## Supplementary information

Supplementary Info

Dataset 1

## Data Availability

All plastic data is uploaded as Supplementary Materials. Discharge data may be obtained from Nicolas Gratiot (nicolas.gratiot@ird.fr).
