# Supplementary material for: Seasonality of riverine macroplastic transport
Source: Sci Rep. 2019 Sep 19;9:13549. doi: 10.1038/s41598-019-50096-1 (PMC6753078; doi:10.1038/s41598-019-50096-1)
Supplement: Supplementary file 1 — Supplementary Info [file 41598_2019_50096_MOESM1_ESM.docx]

**Supplementary materials for “Seasonality of riverine macroplastic transport”**

Tim van Emmerik^1,2^, Emilie Strady^3,4,5^, Thuy-Chung Kieu-Le^3,6^, Luan Nguyen^3^, Nicolas Gratiot^3^

^1^ The Ocean Cleanup, Batavierenstraat 15, 3014JH, Rotterdam, The Netherlands

^2^ Hydrology and Quantitative Water Management Group, Wageningen University, Wageningen, The Netherlands

^3^ CARE, Ho Chi Minh University City of Technology, VNU-HCM, Vietnam

^4^ University of Grenoble Alpes, France

^5^ Aix-Marseille Univ., Mediterranean Institute of Oceanography (M I O), Marseille, Université de Toulon, CNRS /IRD, France

^6^ Faculty of Geology and Petroleum Engineering, Ho Chi Minh City University of Technology, VNU-HCM, Vietnam

**S1: Measurement location**

Fig. S1 indicates the measurement locations of the plastic sampling and counting experiments. The observation sites are numbered from 1 (south) to 12 (north). Plastic samples were primarily taken at locations 2, 3, 10 and 11, as here the highest plastic concentrations were observed.


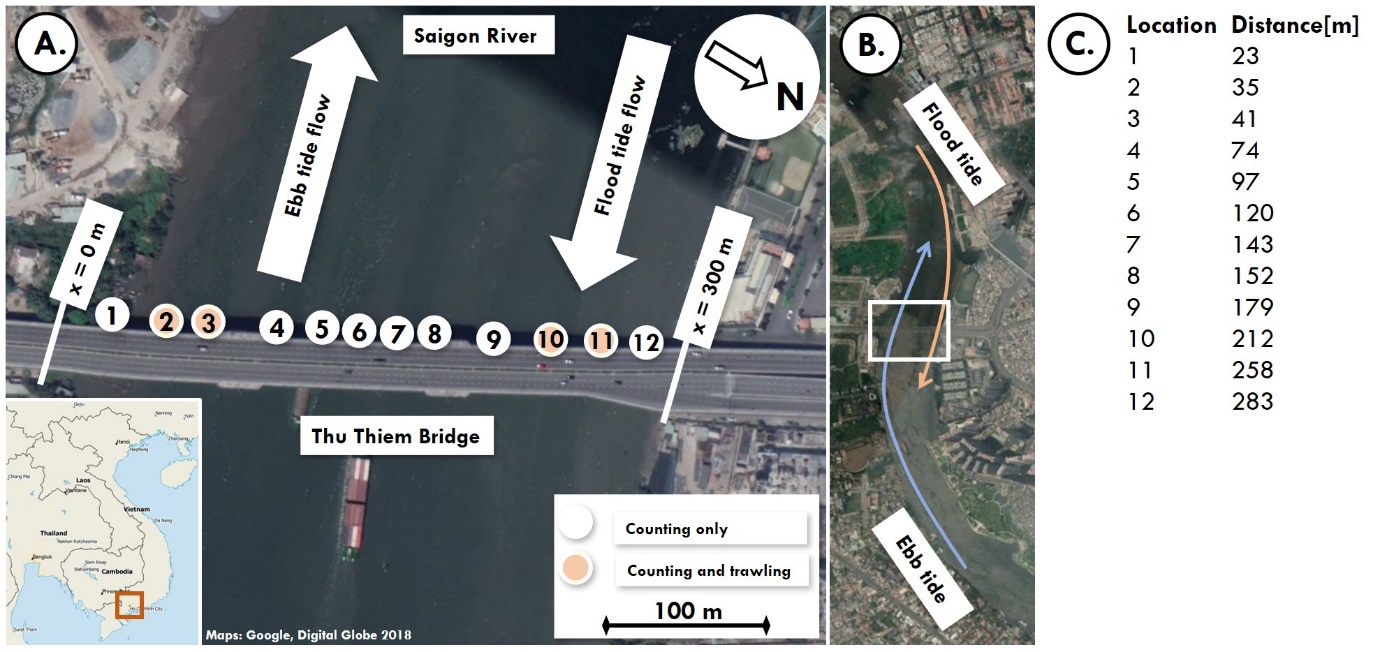


**Figure S1: A. Measurement location and observation sites on the Thu Thiem bridge. Note that the observation sites run from 1 (south) to 12 (north). Arrows project the observed influence of tidal dynamics on plastic flux direction. Bridge-mounted trawls were deployed at location 2 and 3 during ebb tide flow and at location 10 and 11 during flood tide flow (Map: Google, Digital Globe, 2018). B. Overview of the curvature of the river, with an indication of the location of the highest plastic concentration during flood and ebb tides (Map: Google, Digital Globe, 2018). C. Location of each observation point. The figure is reproduced from van Emmerik *et al*. ^1^ (CC BY 4.0).**

**S2: Cross-sectional profiles per month**


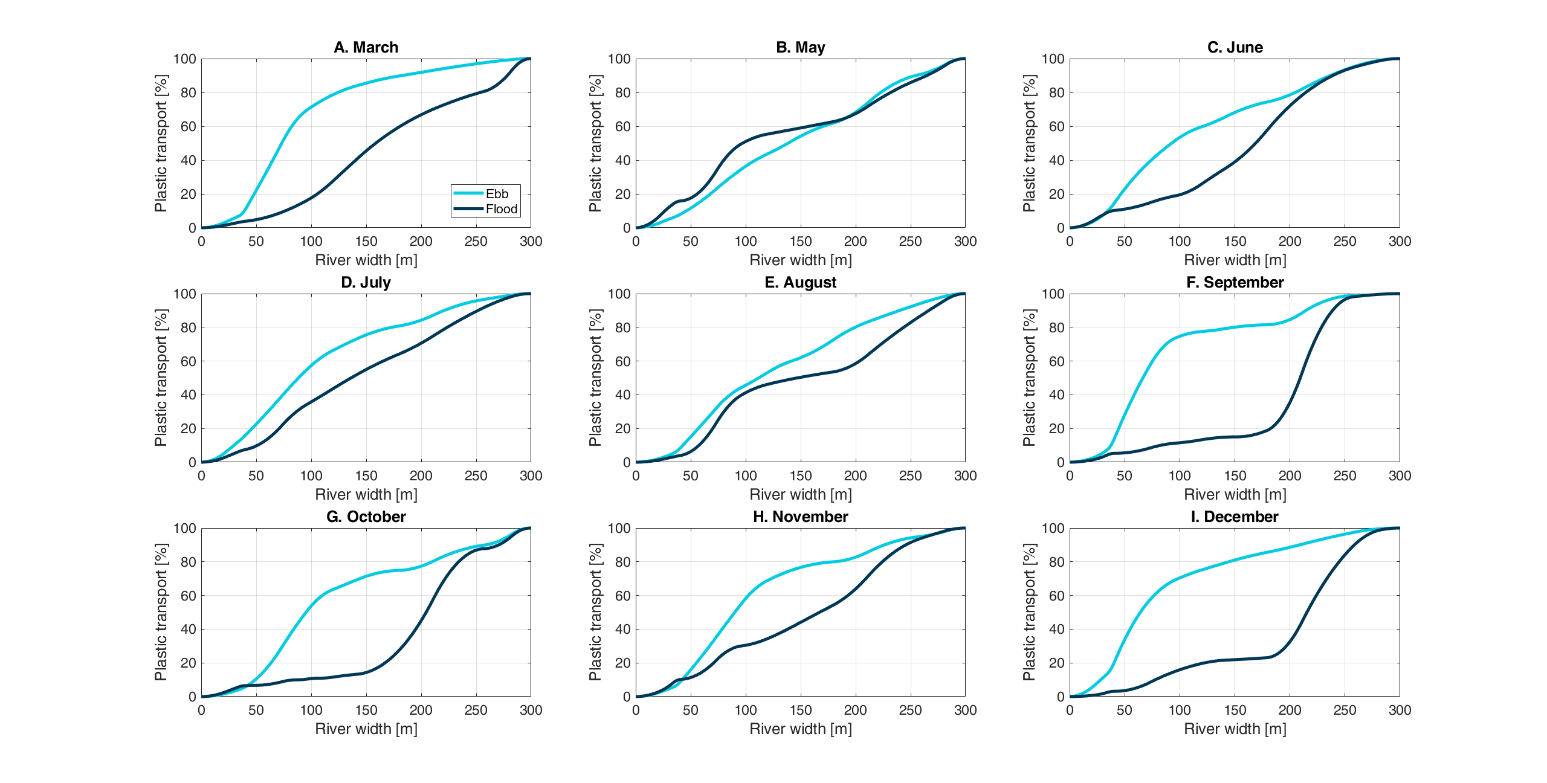


**Figure S2: Cross-sectional distribution of plastic transport during ebb and tide flood for each measured month.**

**S3: Details net sampling**

**Table S3: Sampling days and total sampling duration per month for the net sampling.**

|  | **Month (2018)** | | | | | | | | | | | |
| --- | --- | --- | --- | --- | --- | --- | --- | --- | --- | --- | --- | --- |
|  | J | F | M | A | M | J | J | A | S | O | N | D |
| **Surface only** |  |  |  |  |  |  |  |  |  |  |  |  |
| Sampling days | 0 | 0 | 8 | 0 | 8 | 6 | 13 | 5 | 5 | 12 | 11 | 12 |
| Total sampling duration [hour] | 0 | 0 | 16 | 0 | 6 | 9 | 46 | 22 | 15 | 35 | 18 | 14 |
| **2-layer** |  |  |  |  |  |  |  |  |  |  |  |  |
| Sampling days | 0 | 0 | 0 | 0 | 0 | 0 | 13 | 5 | 5 | 10 | 4 | 0 |
| Total sampling duration [hour] | 0 | 0 | 0 | 0 | 0 | 0 | 96 | 51 | 34 | 57 | 26 | 0 |
| **3-layer** |  |  |  |  |  |  |  |  |  |  |  |  |
| Sampling days | 0 | 0 | 0 | 0 | 0 | 0 | 4 | 0 | 0 | 0 | 0 | 0 |
| Total sampling duration [hour] | 0 | 0 | 0 | 0 | 0 | 0 | 43 | 0 | 0 | 0 | 0 | 0 |

**S4: Details visual counting**

**Table S4: Sampling days, measured profiles and total ebb and flood tide profiles per month for the visual counting measurements.**

|  | **Month (2018)** | | | | | | | | | | | |
| --- | --- | --- | --- | --- | --- | --- | --- | --- | --- | --- | --- | --- |
|  | J | F | M | A | M | J | J | A | S | O | N | D |
| **Visual counting** |  |  |  |  |  |  |  |  |  |  |  |  |
| Sampling days | 0 | 0 | 10 | 0 | 9 | 6 | 14 | 5 | 5 | 11 | 8 | 4 |
| Total measured profiles | 0 | 0 | 82 | 0 | 72 | 64 | 125 | 41 | 31 | 75 | 60 | 28 |
| Ebb tide profiles | 0 | 0 | 48 | 0 | 31 | 40 | 77 | 22 | 25 | 68 | 51 | 25 |
| Flood tide profiles | 0 | 0 | 34 | 0 | 41 | 24 | 48 | 19 | 6 | 7 | 9 | 3 |

**S5: Discharge**


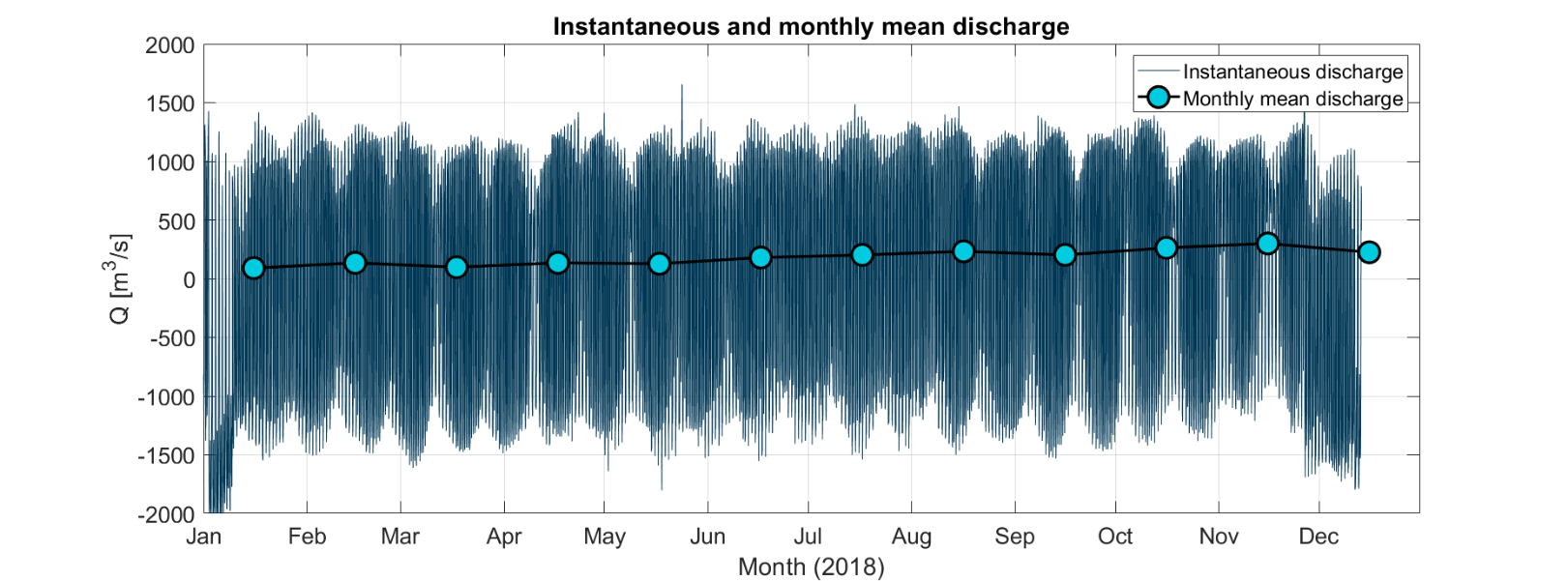


**Figure S5: Instantaneous and monthly mean discharge for the Saigon River in 2018.**

**S6: Plastic transport during ebb tide and flood tide**


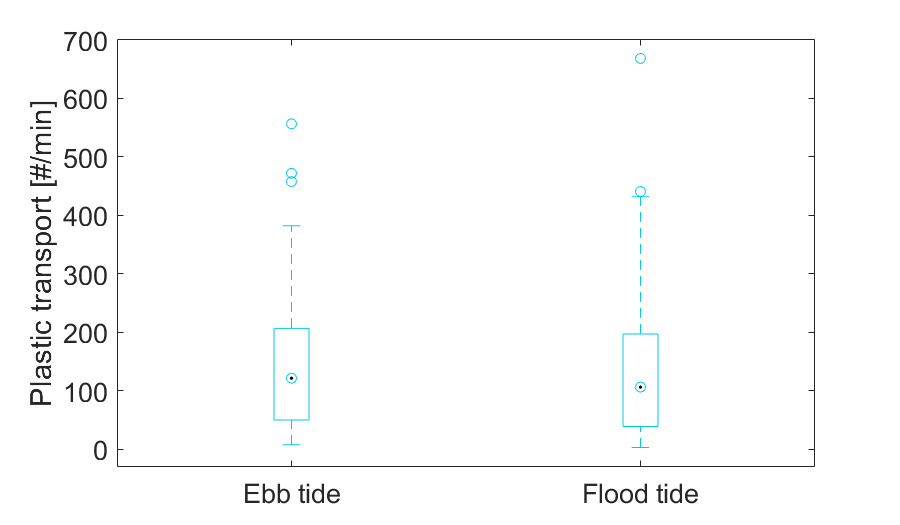


**Figure S6: Boxplots of all ebb tide and flood tide plastic transport measurements.**

**References**

1. van Emmerik, T., *et al*. A Methodology to Characterize Riverine Macroplastic Emission into the Ocean. *Frontiers in Marine Science*, **5**, 372 (2018).
